# Diffuse pulmonary calcification in allergic bronchopulmonary aspergillosis

**DOI:** 10.1016/j.rmcr.2022.101652

**Published:** 2022-04-18

**Authors:** Johnny F. Jaber, Lauran Zeineddine, Divya C. Patel, Diana Gomez-Manjarres

**Affiliations:** a Division of Pulmonary, Critical Care, and Sleep Medicine, University of Florida Health, Gainesville, FL, 32608, USA; b Division of Internal Medicine, University of Florida Health, Gainesville, FL, 32608, USA

**Keywords:** Radiology, Asthma, ABPA, Allergy, Aspergillosis

## Abstract

Allergic bronchopulmonary aspergillosis (ABPA) is a condition that most often occurs in patients with asthma or cystic fibrosis. The diagnosis is usually confirmed by the combination of clinical, radiographic, and immunologic criteria as there is not individual test to establish the diagnosis.

We describe the case of a 64-year-old male with a prior medical history of moderate persistent asthma who presented with worsening cough and was found to have IgE positive for *Aspergillus fumigatus* with findings of diffuse bilateral pulmonary calcifications on HRCT.

## 1 Introduction

Allergic bronchopulmonary aspergillosis (ABPA) is a mixed type I and type III hypersensitivity reaction to the fungus *Aspergillus spp.* that can occur in any structural lung disease, though primarily associated with asthma and cystic fibrosis. The estimated prevalence among asthma patients ranges between 1 and 2% and among patients with cystic fibrosis ranges between 2 and 9%. *Aspergillus spp.* colonization of the airways triggers an IgE, IgG, and Th2 mediated inflammatory response resulting in airway damage and central bronchiectasis. The diagnosis of ABPA is established by demonstrating sensitization to Aspergillus antigens, as well as radiographic findings such as bronchial wall thickening, proximal and central bronchiectasis, nodules, mucus plugging, atelectasis, or ground glass attenuation [1,2]. Diffuse calcifications have been rarely described in ABPA.

## 2 Case presentation

**Fig. 1:**
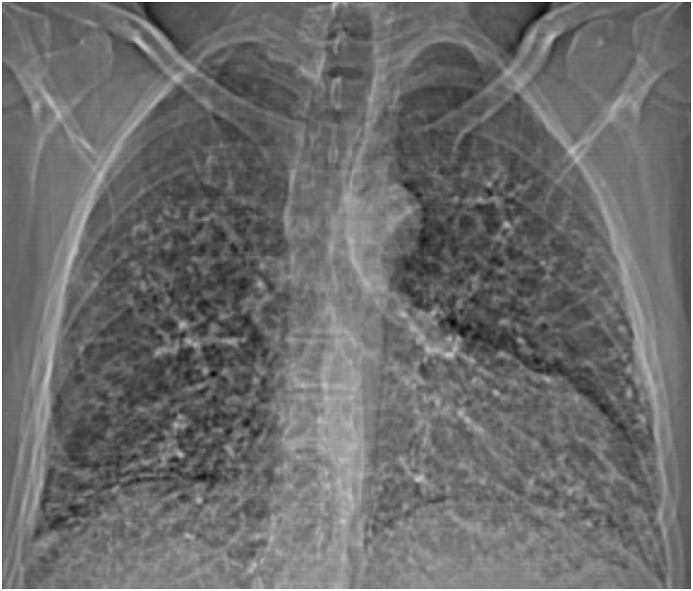
Chest x-ray demonstrating bilateral calcifications and interstitial changes on initial workup.

**Fig. 2:**
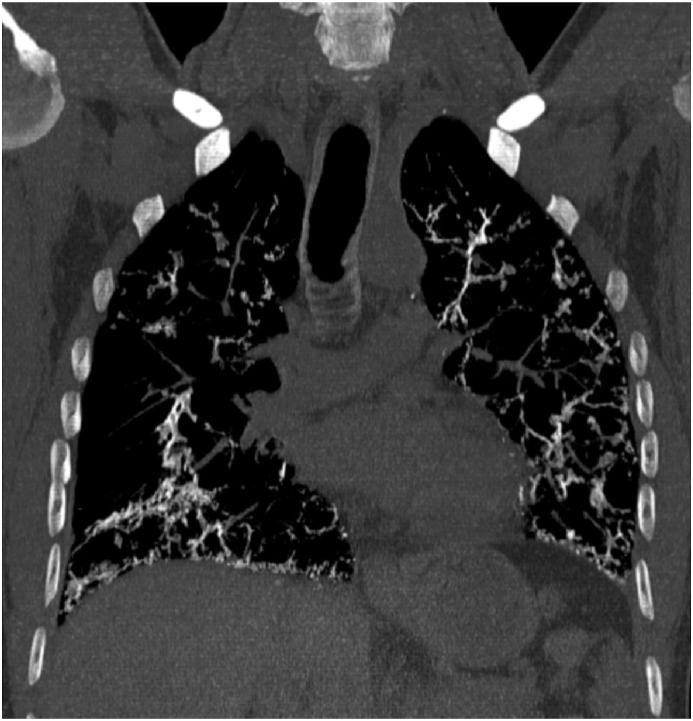
Coronal cut of a high resolution computed tomographic scan of the chest on inspiration using maximal intensity projection using bone windowing demonstrating the diffuse parenchymal calcifications throughout all lobes of the bilateral lungs.

**Fig. 3:**
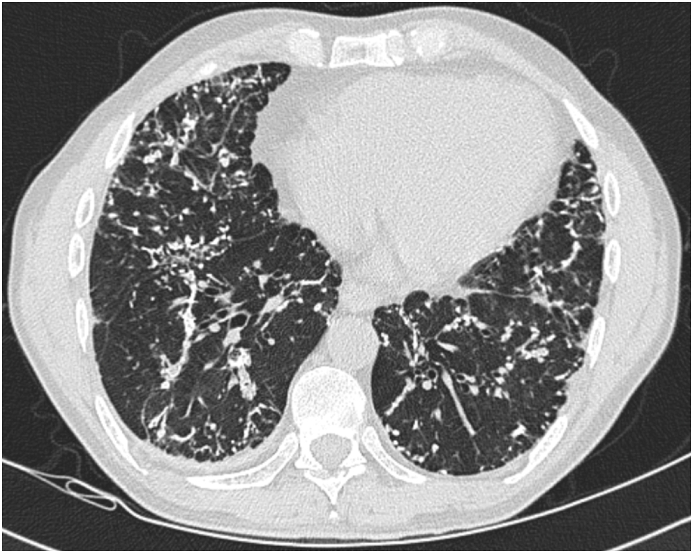
Transverse slice of a high resolution computed tomographic scan of the chest on inspiration with thin slices using lung windowing demonstrating both the bilateral pulmonary opacities, calcifications, and mild bronchiectasis throughout the bilateral lower lobes of the lungs.

**Fig. 4:**
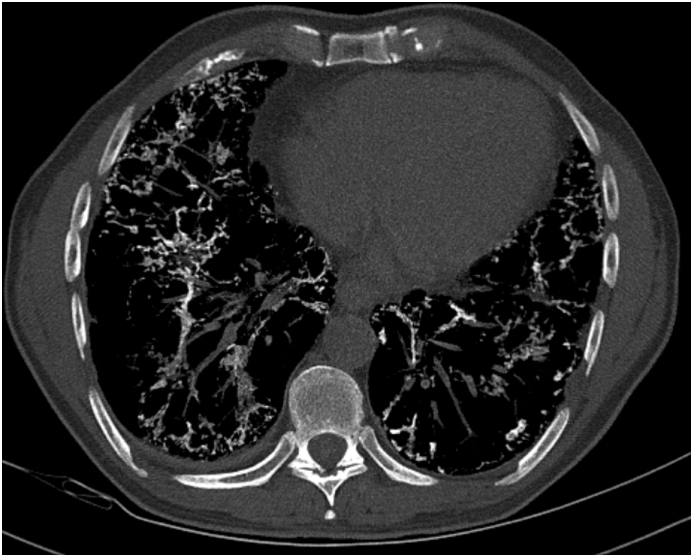
Transverse cut of a high resolution computed tomographic scan of the chest on inspiration using maximal intensity projection using bone windowing demonstrating the diffuse parenchymal calcifications throughout the bilateral lower lobes of the lungs.

A 64-year-old male with a history of moderate persistent asthma and long-standing interstitial lung disease (ILD) classified several years ago as bronchiolitis obliterans with organizing pneumonia (BOOP) presented to general pulmonary clinic with a progressively worsening cough and sputum production. Recently he had been increasing use of his albuterol rescue inhaler to multiple times daily. He previously carried the diagnosis of BOOP from an outside hospital in 1992 incidentally noted on CXR imaging. He was additionally found to be purified protein derivative (PPD) positive at that time and treated with isoniazid for 6 months for latent tuberculosis. He was followed with serial pulmonary function testing and imaging noting stable disease for years prior to establishing care at our clinic. He had an extensive travel history to several African countries. An HRCT of his chest showed diffuse peribronchovascular consolidations with basilar and peripheral predominance, and diffuse perivascular calcifications (Fig. 1, Fig. 2, Fig. 3, Fig. 4). His right lower lobe showed subpleural scarring, and his left lower lobe showed bronchial obstruction with distal reconstitution. Serologic work-up was negative for autoimmune conditions, immunodeficiencies, chronic infections, and cystic fibrosis. Absolute eosinophils were noted to be 1168 cells/uL on complete blood count with differential (CBC). Serum IgE was elevated at 1133 kU/L, with positive IgE for *Aspergillus fumigatus* at 6.12 kU/L. He was diagnosed with allergic bronchopulmonary aspergillosis (ABPA) and started on treatment with two weeks of prednisone 30mg oral daily followed by a six month prolonged taper. Following this taper, the patient reported significant symptomatic improvement and continued his maintenance treatment for asthma and airway clearance. Later surveillance imaging after treatment did not show any significant change in his imaging findings. Serum IgE downtrended to 167 kU/L and peripheral eosinophils decreased to 377 cell/uL with steroid treatment.

## 3 Discussion

While no standardized diagnostic criteria for ABPA exists, widely accepted diagnostic schema include radiographic opacities consistent with ABPA seen on either CXR or HRCT as part of the criteria to establish the diagnosis. Those that meet diagnostic criteria without abnormalities visualized on imaging are labeled as seropositive ABPA (ABPA-S) compared to common presentation ABPA (ABPA-CB). Within those labeled as common presentation, some have proposed further subdivision into mild, moderate, and severe ABPA CB based on radiologic findings [3,4]. Classic findings on CXR in these patients include parenchymal opacities, often in the upper lobes, atelectasis due to mucoid impaction, and findings consistent with bronchiectasis. On HRCT, central bronchiectasis with upper to middle lobe predominance and normal tapering of distal bronchi has been considered a primary feature, however multiple studies have shown varying sensitivity of this finding [1,5]. Other findings that have been commonly described include centrilobular nodular opacities (tree-in-bud), mucoid impaction, parenchymal consolidation and scarring, and pleural effusions or pleural thickening. Up to 30% of all cases can feature high attenuated distal mucoid impaction (HAM), which can also cause bronchocoele formation [3]. The presence or absence of these radiographic findings, particularly the presence of HAM, has been previously proposed to correspond to more severe ABPA as well as risk of recurrence [6]. Our patient presented with both typical and atypical radiographic features of ABPA. There was left lower lobe evidence of central bronchial obstruction with distal tapering. Pulmonary calcifications have traditionally been described in conditions such as occupational lung disease (silicosis, coal workers pneumoconiosis), Dendriform Pulmonary Ossification, metastatic pulmonary calcification, or malignancy. This patient did not endorse such risk factors. Up to 30% of patients have had reported calcifications associated with ABPA thought to be due to long-standing mucoid impaction of the airways, however to our knowledge this is first time such extensive bilateral diffuse calcifications have been associated with this particular pathology [3].

## 4 Conclusions

The diagnosis of ABPA is based on several factors, most importantly dependent serologic and radiologic domains. In those with a predisposing condition such as asthma or cystic fibrosis, both serologic evidence of sensitivity to *Aspergillus spp.* as well as compatible radiographic findings are needed to confirm the diagnosis. Common radiographic findings consistent with the diagnosis of ABPA include central bronchiectasis, parenchymal scarring, and mucoid impaction. Though occasional calcifications can be seen, diffuse and extensive bilateral pulmonary calcifications have not previously been described in those with ABPA. The patient we have described had both serologic and other radiographic findings compatible with underlying ABPA, including an appropriate response to therapy, making ABPA the most likely compatible etiology of his calcifications.

## Author participation

Authors JJ, LZ, DG, and DP all participated equally in the creation of this manuscript and have made substantial contributions to this work.

## Declaration of competing interest

On behalf of all authors, the corresponding author declares that there are no conflicts of interest.
